# The influence of different patient positions during rapid induction with severe regurgitation on the volume of aspirate and time to intubation: a prospective randomised manikin simulation study

**DOI:** 10.1186/s12871-019-0686-x

**Published:** 2019-01-24

**Authors:** Michael St. Pierre, Frederick Krischke, Bjoern Luetcke, Joachim Schmidt

**Affiliations:** Anästhesiologische Klinik, Universitätsklinikum Erlangen, Krankenhaustrasse 12, 91054 Erlangen, Germany

**Keywords:** Airway management, Aspiration, Rapid sequence induction, Patient safety, Simulation

## Abstract

**Background:**

Aspiration is a main contributor to morbidity and mortality in anaesthesia. The ideal patient positioning for rapid sequence induction remains controversial. A head-down tilt and full cervical spine extension (Sellick) might prevent aspiration but at the same time compromise airway management. We aimed to determine the influence of three different positions during induction of general anaesthesia on the volume of aspirate and on participants’ airway management.

**Methods:**

Eighty-four anaesthetic trainees and consultants participated in a prospective randomised simulation study. Anaesthesia was induced in reverse Trendelenburg position (+ 15°) in a manikin capable of dynamic fluid regurgitation. Participants were randomised to change to Trendelenburg position (− 15°) a) as soon as regurgitation was noticed, b) as soon as ‘patient’ had been anaesthetised, and c) as soon as ‘patient’ had been anaesthetised and with full cervical spine extension (Sellick). Primary endpoints were the aspirated volume and the time to intubation. Secondary endpoints were ratings of the laryngoscopic view and the intubation situation (0–100 mm).

**Results:**

Combining head-down tilt with Sellick position significantly reduced aspiration (*p* < 0.005). Median time to intubate was longer in Sellick position (15 s [8–30]) as compared with the head in sniffing position (10 s [8–12.5]; *p* < 0.05). Participants found laryngoscopy more difficult in Sellick position (39.3 ± 27.9 mm) as compared with the sniffing position (23.1 ± 22.1 mm; *p* < 0.05). Both head-down tilt intubation situations were considered equally difficult: 34.8 ± 24.6 mm (Sniffing) vs. 44.2 ± 23.1 mm (Sellick; *p* = n.s).

**Conclusions:**

In a simulated setting, using a manikin-based simulator capable of fluid regurgitation, a − 15° head-down tilt with Sellick position reduced the amount of aspirated fluid but increased the difficulty in visualising the vocal cords and prolonged the time taken to intubate. Assessing the airway management in the identical position in healthy patients without risk of aspiration might be a promising next step to take.

## Background

Pulmonary aspiration has been a feared complication of anaesthesia from the very start. Currently, the rapid (sequence) induction and intubation is the technique of choice for securing the airway in patients at risk of aspiration [1–3]. However, despite the technique’s widespread use, there is still an ongoing debate concerning the quality of evidence supporting its use [4], as well as the components and execution of the technique [3, 5]. In particular, the ideal positioning of the patient at risk of regurgitation and aspiration at the time of induction remains controversial [3, 6]. Historically, a semi-sitting head-up position with a pillow under the occiput [7], a supine position with a slight head-down tilt [8], as well as the head-down position [9] have been suggested. Despite the controversy surrounding the induction, it is sometimes recommended to tilt the patient head-down as soon as an aspiration event during induction has occurred [6, 10, 11].

A few years ago Takenaka and colleagues used an airway management trainer as a static pulmonary aspiration model to determine the optimal head-down tilt and head–neck positions for preventing aspiration[12]. Their results suggest that only a head-down tilt (≥ 10°) combined with a full cervical spine extension (Sellick) was suitable to prevent aspiration within a clinically relevant range. They qualified their statement by pointing out that this position may not be the best for laryngoscopy.

Therefore, we wanted to see how the reported benefits of a head-down tilt combined with a full cervical spine extension might translate into a rapid sequence induction in a dynamic model of a regurgitating patient.

Using the design specifications of the static pulmonary aspiration model [12] as a starting point, we developed a manikin-based simulator capable of dynamic fluid regurgitation. The two primary objectives of this study were to a) determine the influence of three different positions during induction of general anaesthesia on the volume of aspirate in the manikin’s trachea and bronchi when severe regurgitation occurred and b) the mean time to intubate in the two final head positionings (Trendelenburg position with sniffing position and Trendelenburg with Sellick extension).

The secondary objectives were participants ratings of the difficulty in visualising the vocal cords and the difficulty of the head-down tilt intubation situation, partly with full cervical spine extension.

## 1Methods

### Participants

**Fig. 1:**
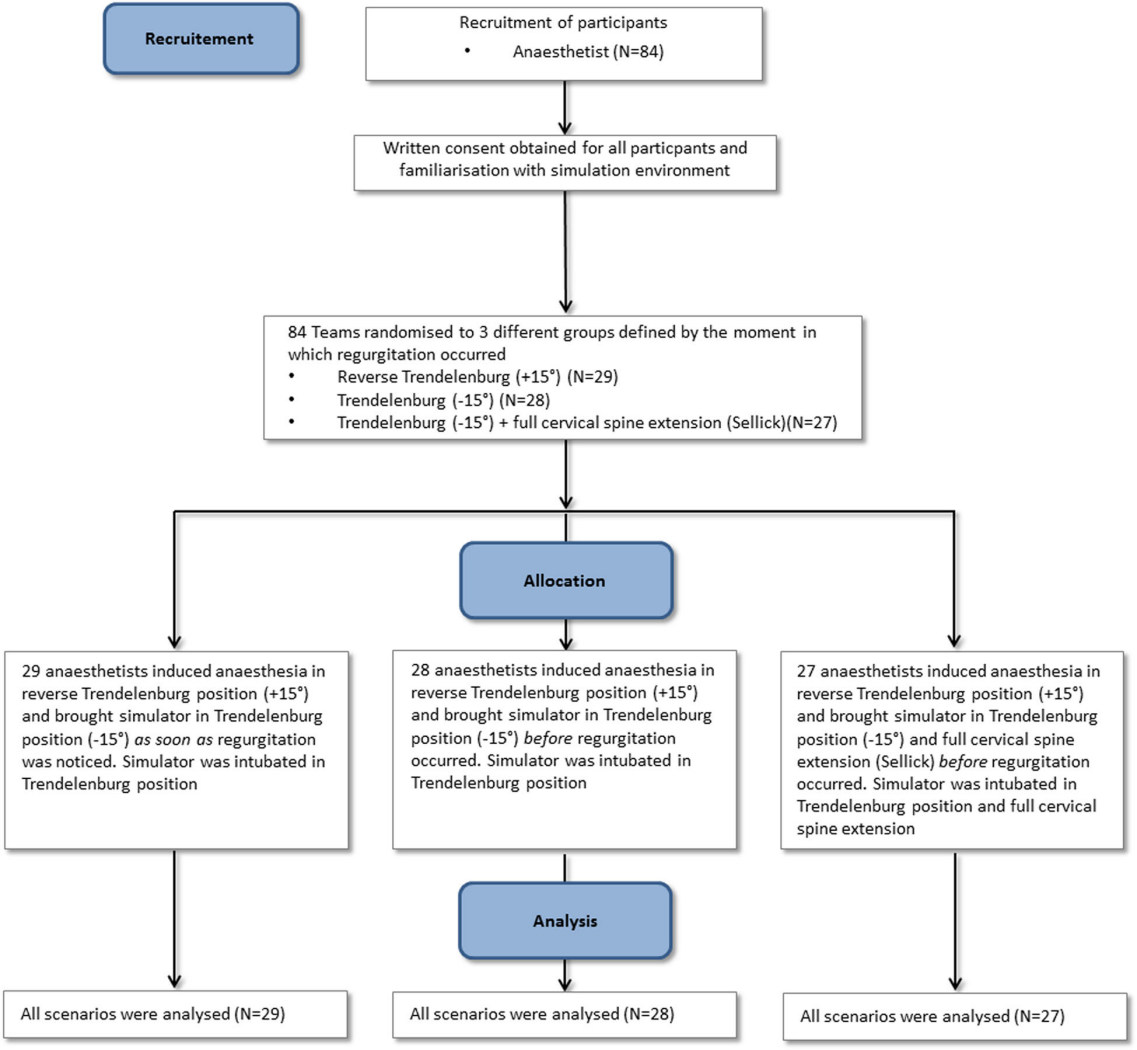
CONSORT flow chart of recruitment, randomisation, and analysis

After obtaining approval of the study protocol by the ethics committee of the Friedrich-Alexander University Erlangen-Nuremberg (reference number 002_18 B), we enrolled 84 participants into this prospective, randomised controlled trial (Fig. 1). Participants were part of the annual institutional simulation training programme at the authors’ department (February – March 2018). Written informed consent was obtained from all participants prior to the scenario.

### Study protocol

**Fig. 2:**
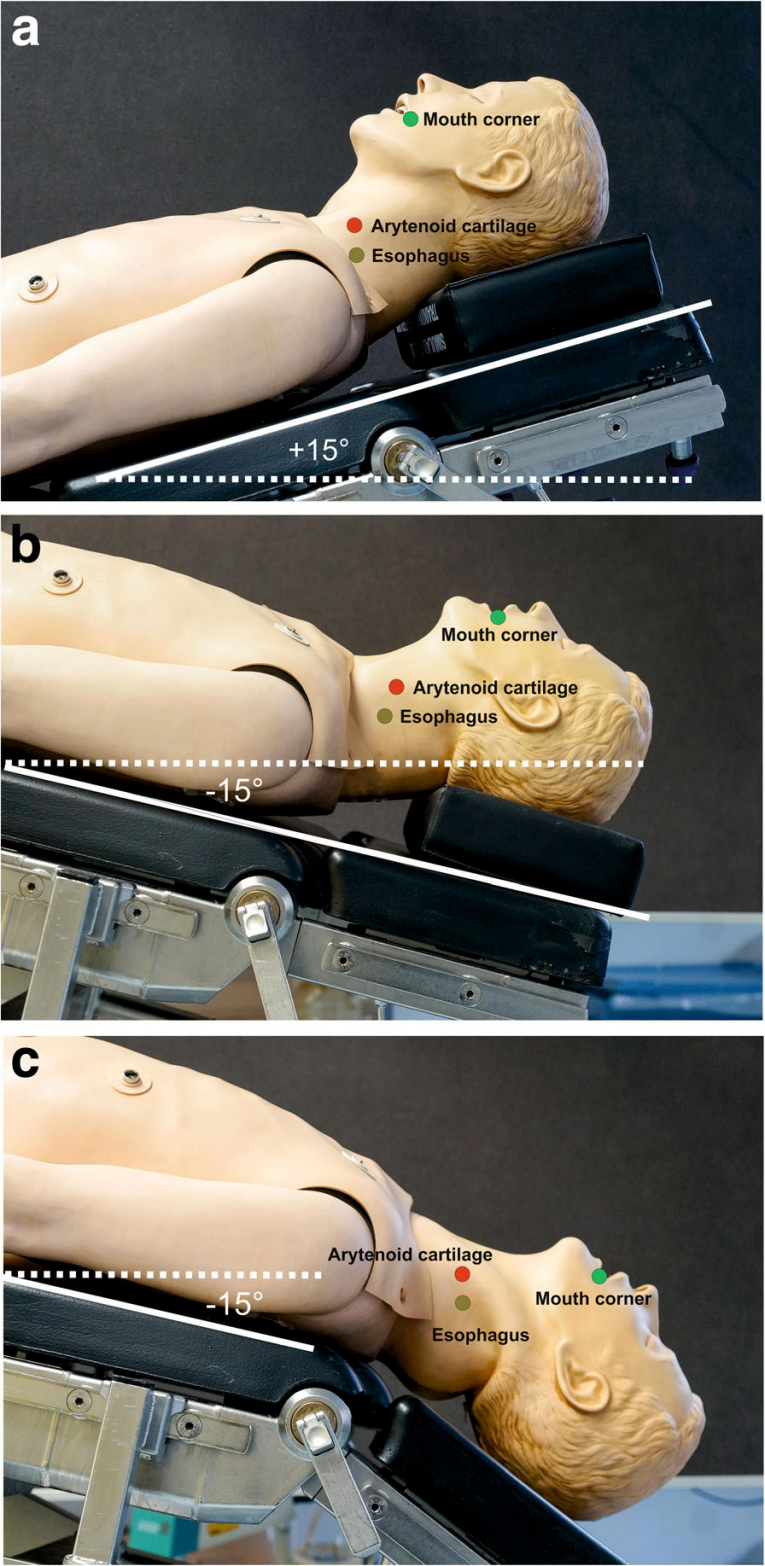
The three positions of the manikin used: (**a**) Reverse Trendelenburg position (+ 15°) and head supported by a pillow (‘sniffing position’); (**b**) Trendelenburg position (− 15°) and ‘sniffing position’; (**c**) Trendelenburg position (− 15°) and full cervical spine extension (‘Sellick’). Sellick position was determined by tilting the head rest until the head started to suspend in mid-air

We did not perform an a priori sample size calculation, but used a convenience sample, targeting all participating consultants and anaesthetic trainees. Using a web-based tool (https://www.randomizer.org) participants were randomly assigned to one of three groups which were defined by a) the position in which regurgitation occurred and b) the presence or absence of full cervical spine extension (Sellick-position; Fig. 2):

Group 1: Induction of anaesthesia in reverse Trendelenburg position (+ 15°). As soon as regurgitation occurred the mannekin was placed in Trendelenburg position (− 15°) and intubated in Trendelenburg position with the head supported by a pillow (‘sniffing position’).

Group 2: Induction of anaesthesia in reverse Trendelenburg position (+ 15°). As soon as the ‘patient’ had been anaesthetised the mannekin was placed in Trendelenburg position (− 15°). Thereupon regurgitation occurred and the simulator was intubated in Trendelenburg position with the head supported by a pillow (‘sniffing position’).

Group 3: Induction of anaesthesia in reverse Trendelenburg position (+ 15°). As soon as the ‘patient’ had been anaesthetised the mannekin was placed in Trendelenburg position (− 15°) and full cervical spine extension (Sellick). Thereupon regurgitation occurred and the simulator was intubated in Trendelenburg position without the pillow supporting the head and with full cervical spine extension.

The extent of full cervical spine extension was determined by positioning the manikin’s neck over the joint of the headrest and then tilting the headrest until the head started to suspend in mid-air. The two positions for reverse Trendelenburg and Trendelenburg position were defined by the maximum tilt of the mobile OR-table (range: + 15° to − 15°) used at our hospital.

Participants from all three groups were briefed on the scenario with a scripted explanation of the purpose and methodology of the study. The script was supplemented with a final passage in which participants received a detailed description on how to proceed with the allocated induction protocol.

Participants were asked to perform a rapid sequence induction in a patient with a suspected acute mesenteric infarction. Once anaesthesia had been induced, the patient was positioned according to the randomisation protocol. Every participant expected to receive a verbal cue when to start with laryngoscopy and was surprised when the mannekin regurgitated fluid instead.

Simulations were run by combining a modified resuscitation manikin (HeartSim 4000; Laerdal Norway), capable of regurgitating liquid, with the monitor display of a manikin-based simulator (SimMan; Laerdal Norway).

Haemodynamic and pulmonary variables were programmed as trends into the software of the SimMan. To add time pressure to the induction, the rate of desaturation during apnoea was approximated to a published time course of desaturation in apnoea in obesity [13]. Regurgitation was triggered by the injection of the muscle relaxant with a 20 s delay. Study protocol allowed participants to manage regurgitation at their own discretion (i.e. intubation during regurgitation with simultaneous suctioning or after regurgitation had ceased).

### Modification of a resuscitation manikin

**Fig. 3:**
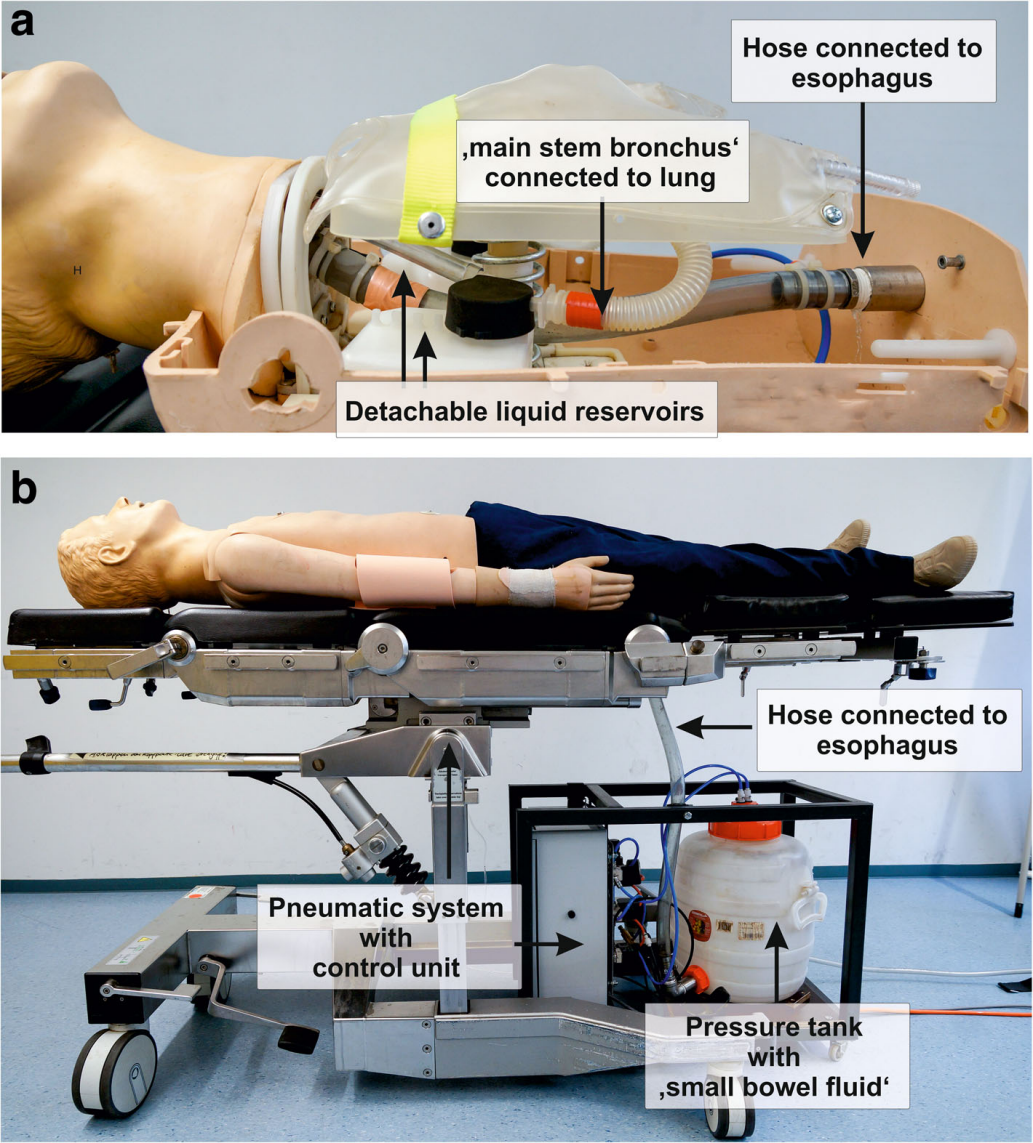
Setup of the manikin: (**a**) The tracheal tree was modified by inserting two detachable reservoirs that collected the aspirated fluid while leaving ventilation unchanged. The oesophagus was connected to the pneumatically driven liquid reservoir with a flexible hose; (**b**) Operational manikin. During simulations, the pneumatic system and liquid reservoir were hidden from the participants by surgical drapes

The resuscitation manikin was equipped with an airway identical to that used in the static pulmonary aspiration model [12]. The manikin’s bronchi were modified with two detachable reservoirs, which collected the regurgitated fluid and allowed quantification of the aspirated volume (Fig. 3). To simulate an elevated intragastric pressure we developed a pneumatic system with a pressure-stable reservoir containing the ‘small bowel liquid’. The liquid reservoir was connected to the manikin’s esophagus through a large riser 4 cm in diameter. A memory-programmable control controlled two pneumatic 2/3 directional control valves (V1, V2). On activation, both valves allowed pressurised air to flow into the pressure tank, consecutively displacing the fluid towards the manikin. One second after the liquid had reached the hypopharynx and had created an initial surge, the system turned off V1 and continued to provide a pressure of 25 mbar (18.75 mmHg) via V2, which provided a constant diminished flow of 8 s duration. We chose a pressure of 25 mbar because intra-abdominal measurements have shown that severe abdominal infections can generate pressures up to > 20 mmHg [14]. The dynamics of the regurgitation as well as the amount of regurgitated fluid appeared to be clinically realistic to all four authors. ‘Small bowel fluid’ was created by mixing water with soluble coffee powder and vinegar in a predefined ratio. Finally, the dynamics of the regurgitation, as well as the haemodynamic and pulmonary trends, were pretested before study commencement using nonparticipating subjects, and the results served to refine both simulation components.

### Data collection

After each scenario the quantity of liquid in the intrapulmonary reservoirs was measured. Multiscreen synchronised video recordings were available for offline evaluation. Time taken to intubate was defined as the interval between the moment the participant started to open the mouth to introduce the laryngoscope and the insertion of the orotracheal tube.

At the end of the simulation session participants were asked to rate the subjective ease in visualising the vocal cords and the difficulty of the head-down tilt intubation situation, partly with full cervical spine extension, on a visual analogue scale (0–100 mm: 0 = not difficult; 100 = worst possible difficulty).

### Statistics

Data were analysed with the use of SPSS software version 21.0 (IBM). Homogeneity of variances was assessed with Levene’s test. Where variance across groups was equal (e.g. participant characteristics, survey ratings, mean volumes of aspirate) the independent sample t-test and ANOVA were utilised. Where homogeneity of variance was violated (e.g. time to intubation) a non-parametric equivalent of the analysis was conducted (Welch’s unequal variance t-test). A Spearman’s rank-order correlation was run to determine the relationship between years of clinical experience and the time taken to intubate, the difficulty in visualising the vocal cords, and the difficulty of the intubation situation in all three groups. Visual analogue scale ratings as well as other parametric data are reported as mean ± SD. Non-parametric data are presented as the median and interquartile range [IQR]. All reported *p*-values are two-sided, and *p*-values of less than 0.05 were considered statistically significant.

## Results

**Fig. 4:**
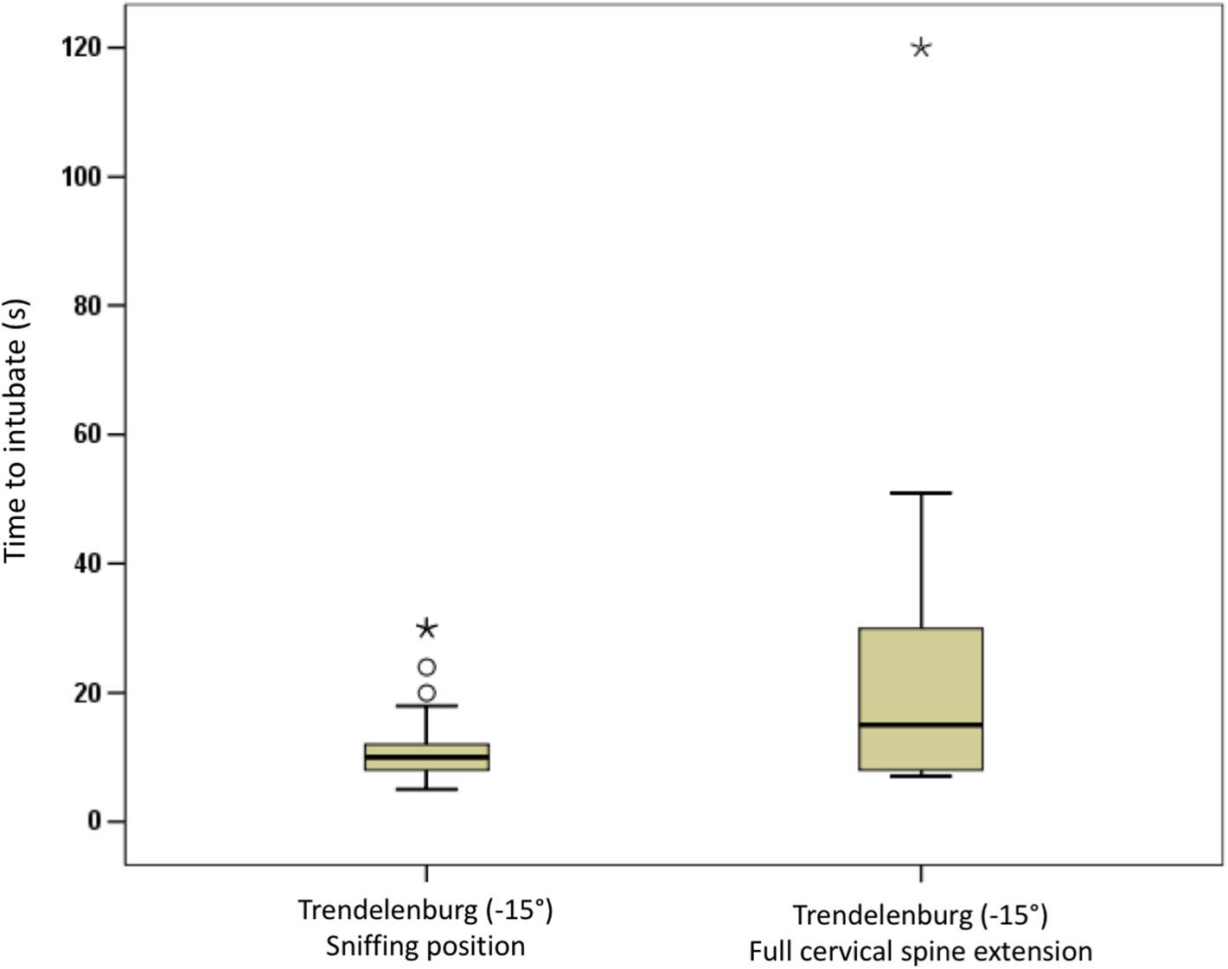
Intubation position and the resulting time to intubate. Intubation times ranged from 5 to 30 s in the sniffing position as compared to 7 to 120 s in full cervical spine extension

Eighty-four anaesthetic trainees and consultants participated in the study (Fig. 1). There were no group differences in terms of years of clinical experience (Table 1). All participants intubated the manikin after regurgitation had ceased. There was a significant difference in mean volume of aspirate as a function of positioning during induction: 588 ± 157 ml vs. 414 ± 224 ml for group 1 vs. group 2 (*p* < 0.005) and 414 ± 224 ml vs. 43 ± 59 ml for group 2 vs. group 3 (*p* < 0.005) (Fig. 4). In group 3, the bronchial tree and the reservoirs were completely free of aspirated liquid in 48% of cases (13 of 27).

**Table 1:** Participant characteristics of the randomly assigned groups: sex, age and years of clinical experience

Characteristics	Reverse Trendelenburg (+ 15°) *(n = 29)*	Trendelenburg (− 15°) *(n = 28)*	Trendelenburg (− 15°) + Sellick *(n = 27)*	*p*
Sex (m:f)	17:12	10:18	16:11	n.s.
Age (yrs)	33.1 (± 6.1)	37.0 (± 7.2)	36.1 (± 8.5)	n.s.
Clinical Experience (yrs)	6.8 (± 5.5)	8.1 (± 6.3)	8.6 (± 7.0)	n.s.

Participants were randomly assigned to one of three groups which were defined by the position in which regurgitation occurred (Reverse Trendelenburg or Trendelenburg) and the presence or absence of full cervical spine extension (Sellick-position) in the Trendelenburg group

Data are mean (±SD)

Median time to intubate was longer when the head was in full cervical spine extension (group 3) as compared with the head in sniffing position (groups 1 and 2: 15 s [8–30] vs. 10 s [8–12.5]; *p* < 0.05). Intubation times ranged from 7 to 120 s in full cervical spine extension as compared with 5 to 30 s in the sniffing position (Fig. 3).

Participants found it more difficult to visualise the vocal cords during full cervical spine extension (group 3) as compared with the sniffing position (groups 1 + 2: 39.3 ± 27.9 mm vs. 23.1 ± 22.1 mm; *p* < 0.05).

Both head-down tilt intubation situations were considered equally difficult: 34.8 ± 24.6 mm (groups 1 + 2) vs. 44.2 ± 23.1 mm (group 3; *p* = n.s).

The years of clinical experience correlated weakly with the time to intubation (groups 1 + 2: r_s_(57) = − 0.19, *p* = n.s; group 3: r_s_(27) = − 0.18, *p* = n.s), with participants’ rating of the difficulty in visualising the vocal cords (groups 1 + 2: r_s_(57) = − 0.24, *p* = n.s; group 3: r_s_(27) = − 0.22, *p* = n.s), and with their appraisal of the head-down tilt intubation situation (groups 1 + 2: r_s_(57) = − 0.32, *p* = n.s; group 3: r_s_(27) = − 0.37, *p* = n.s).

## Discussion

Patient positioning during induction and intubation in patients with risk of aspiration has been reviewed in several recent publications [15–18]. Although the head-down (Trendelenburg) position has been advocated together with the supine position and the semi-sitting position as one of three preventive methods against aspiration during rapid induction and intubation for more than 50 years [9] there is little data on the feasibility of such a technique. In a recent German survey reporting on patient positioning in the clinical setting, the majority of respondents preferred a semi-sitting (84%) or a supine (13%) positioning, while the induction in Trendelenburg position was chosen by 3% of anaesthetists [19]. Another German study addressing rapid sequence induction in the prehospital setting found a comparable frequency distribution: the preferred positioning was a semi-sitting (61,8%), followed by a supine (33%), and a moderate Trendelenburg-position (3,5%) [16].

The debate about the ideal position centres around five clinical aspects: the likelihood of passive regurgitation, the inevitability of aspiration, the quality of preoxygenation, the difficulty in securing the airway, and haemodynamic sequelae.

Proponents of the *head-up position* argue that preoxygenation is improved in both obese and non-obese patients [1, 17, 20]. Furthermore, the likelihood of regurgitation is reduced because the gravitational effect of the head-up position may exceed the intragastric pressure [2, 3]. However, if regurgitation does occur, gastric content may readily enter the trachea and bronchi before the anaesthetist becomes aware of the condition [8].

Supporters of the *supine position* contend that the anaesthetist’s familiarity with this position will accelerate endotracheal intubation and that research has provided convincing evidence that regurgitation in the supine position can be prevented as long as cricoid pressure is applied properly [21].

The main advantage of a *head-down position* is that the carina is higher than the larynx and that regurgitated matter does not find its way into the tracheobronchial tree [12]. The main concern with the Trendelenburg position has always been that it may actually predispose to regurgitation by increasing gastric pressure [2, 3]. However, regurgitation may not be a concern in patients without gastrointestinal pathology. In awake, non-fasting volunteers, a change in body position (20° head-up, supine, 20° head-down) had no influence on the frequency of gastroesophageal reflux or on the intragastric-oesophageal barrier pressure [22]. In a similar vein, a 15° head-down tilt caused no change in intragastric and barrier pressure in patients undergoing gynaecologic surgery [23]. In obese patients, too, the intragastric-oesophageal barrier pressure remained positive in the Trendelenburg position (− 20°) [24]. Yet, these results may not apply to patients suffering from acute abdomen conditions with an elevated intra-abdominal pressure. For this reason, we chose acute mesenteric infarction as the clinical scenario for our study. Finally, the Trendelenburg position has been associated with an increased risk of hypoxia, as airway management may be more difficult and may take longer [2, 3]. Furthermore, reduced lung compliance may impair effective mask ventilation should this become necessary [2, 3].

In the attempt to prevent aspiration completely, Takenaka [12] suggested modifying the Trendelenburg position by combining the head-down tilt with the Sellick position [8]. In this position, the height of the corner of the mouth will be lower than the arytenoid cartilage and tracheal bifurcation, allowing liquid to flow freely away from the larynx. However, without the head supported by a pillow the resulting anatomical position has the potential to make tracheal intubation more difficult.

In our study, the mean volume of aspirate was the highest in the group where the manikin was placed head down only after regurgitation had started. These findings corroborate the concern that during passive regurgitation gastric content will enter the trachea and bronchi before the anaesthetist becomes aware of the condition.

The mean volume of aspirate was less in the Trendelenburg position and even less when combined with the Sellick position. In contrast with Takenaka’s study, in which a 10° head-down tilt with Sellick position completely prevented aspiration [12], the identical position prevented aspiration in only about half of our cases. We explain this discrepancy by the slightly differing head-down tilt angles in both studies. Whereas Takenaka achieved maximum neck extension by taping the manikin’s head to the table, our study protocol required that neck extension stopped as soon as the head started to suspend in mid-air. As a result, the mouth corner might not always have levelled with the arytenoid cartilages.

Placing the simulator in the Trendelenburg position with an elevated head did not delay intubation as compared with the conventional supine position. Our measured intubation times (ranging from 5 to 30 s) are consistent with reported intubation times of 9–30 s when a Macintosh laryngoscope was used in an airway management trainer [25–28].

However, placing the head in full cervical spine extension significantly prolonged mean intubation times from 10 s to 15 s. It is arguable whether this difference of 5 s, despite statistical significance, has any clinical relevance. Clinically relevant, however, is the fact that 5 out of 27 participants (18.5%) needed between 40 and 120 s until the airway was secured (Fig. 3). In a clinical setting with a small bowel ileus, this delay would most certainly put the patient at risk of hypoxia. As presumed, participants found it more difficult to visualise the vocal cords in Sellick position than in the familiar sniffing position. Because no participant had ever intubated a manikin or a patient in Sellick position prior to the study we cannot exclude the possibility that unfamiliarity with the approach rather than genuine anatomical problems account for the difference. Given the learning curve for intubation [29] it is conceivable that repeated intubations in head-down tilt with Sellick position would shorten intubation times and possibly eliminate statistical outliers. If that were the case, the Trendelenburg position with full cervical spine extension would indeed offer protection from aspiration without putting the patient at an unjustifiable risk of hypoxia. In any case, in order to become solid clinical skills, both head-down intubating positions would have to be trained on a regular basis in healthy patients with a normal airway.

Our finding that a head-down tilt (− 15°) with full cervical spine extension can prevent pulmonary aspiration raises the clinical question whether intubation should be delayed until the end of regurgitation with the patient placed in Trendelenburg position or whether the upper airway should be protected as quickly as possible with the aid of a large bore suction. Our preliminary findings in a simulated setting offer the clinician another strategy to consider in case of regurgitation but do not warrant any final conclusion. Rather, this decision will continue to depend upon the anaesthetist’s situational assessment of the volume of regurgitating fluid, the dynamic of the regurgitation (continuous oesophageal rise or one short surge) and the effectiveness of the suctioning efforts.

In the setting of aspiration, regurgitation occurs more commonly than active vomiting [30]. General anaesthetic techniques attenuate the protective upper airway reflexes and physiological mechanisms that prevent regurgitation and aspiration. Excessively light depths of anaesthesia in combination with insufficient neuromuscular blockade may evoke gastrointestinal motor responses during laryngoscopy and intubation such as gagging or retching that may increase gastric pressure over and above lower oesophageal sphincter pressure facilitating regurgitation [31]. Allthough study protocol left the choice of drugs at the discretion of the anaesthetist, all participants followed local clinical guidelines for rapid sequence induction which included fentanyl, an induction agent (e.g. etomidate, propofol, or thiopental), and rocuronium as non-depolarizing neuromuscular blocking agent which would have provided an adquate depth of anaesthesia and complete neuromuscular blockade in a real patient.

### Limitations of the study

The results of this simulation study indicate that the rapid induction and intubation in Trendelenburg position with full cervical spine extension might be a feasible option to prevent aspiration and to secure the airway in a justifiable time. However, the findings cannot simply be transferred to the clinical context for several reasons.

First, a modified manikin was used as a model for pulmonary aspiration. Although relevant anatomical landmarks in airway trainers (e.g. arytenoid cartilage, oesophagus, mouth corner) may correlate with landmarks in adult human volunteers [12], extrapolating results from the evaluation in manikins to humans is nevertheless problematic. A comparison of computed tomography scans of patients and airway trainers revealed that the pharyngeal airspace of airway trainers is generally wider than a patient’s pharyngeal airspace [32]. As a result, the wide pharyngeal airspace could lead to an inappropriately easy airway. This structural feature of airway trainers might explain the fact that we were unable to observe a correlation between years of clinical experience and time to intubate: the airway made intubation easy for both, novices and experts. Furthermore, a single airway trainer does not reflect the multitude of different airways found in real patients. Hence, the time to intubate an airway trainer can be significantly different from the time taken to insert an airway device in humans [33].

Second, the synthetic laryngeal structure of the airway trainer with the vocal chords fixated in abducted position created an unobstructed inflow tract for the regurgitating fluid. Assuming that a volume of 0.8–1 ml/kg (or > 50 ml in a 70 kg adult) with a pH < 3,5 is the critical value for the development of an aspiration pneumonitis [34], this value would have been exceeded in 75% (63/84) of measurements. However, we do not want to draw any clinical conclusions, but rather wish to interpret the trend towards lower mean volumes in the head-down positions as an indication that patients at risk of aspiration may benefit from a Trendelenburg position in combination with a Sellick position. After all, 48% of manikins in group 3 did not experience aspiration and when they did, the mean aspirated volume amounted to 1/15th and 1/10th of groups 1 and 2, respectively.

Third, our model can only simulate the clinical condition of passive aspiration following gravity. However, the clinical consequences of regurgitation may be more severe in a spontaneously breathing patient where respiration efforts may actively suction regurgitated fluid into the airways, hereby increasing the volume of aspirate.

Fourth, the Trendelenburg position may not be an option in obese patients in which an additional head-down position will further reduce functional residual capacity, thereby increasing the risk of hypoxia. Finally, a full cervical spine extension will not be feasible in patients with reduced cervical spine mobility or suspected cervical spine injury.

A major limitation of our study is the fact that it was not conducted as a separate clinical study but instead part of our annual institutional simulation training programme. Time and ressources available only permitted one rapid sequence induction per participant which possibly introduced a bias in each group about experience and technical skills. A cross-over study with the repetition of the three situations for every participant with a randomized order in the groups might have avoided this possibility and would have increased the power of our study.

Normally, manikin studies conclude their discussions by pointing to the fact that further clinical studies are needed to evaluate the results in patients. In an editorial on the uncontrolled proliferation of manikin studies in evaluating new airway equipment, the authors demanded that researchers should follow up their published manikin-based study with a patient-based study, to confirm the initial results [33]. This claim, however, leaves us at an impasse. A follow-up study in patients at risk of aspiration is out of question for ethical reasons. As a result, we do not know how the observed benefits of the two Trendelenburg positions in general, and the Trendelenburg position with full cervical spine extension in particular, could possibly translate into clinical practice, especially as airway management should be informed by the respective national airway management guidelines. Unfortunately, until now few national societies have issued recommendations on rapid-sequence inductions. If they have, they did not usually address the issue of patient positioning [5, 35].

However, assuming that a head-down tilt (− 15°) with full cervical spine extension can prevent pulmonary aspiration, a question worth pursuing might be how the simulated laryngoscopic view and intubation situation correlate with an identical airway management in healthy patients who have no risk of aspiration and who can be effortlessly mask-ventilated.

## Conclusions

In a simulated setting, using a manikin-based simulator capable of dynamic fluid regurgitation, the rapid sequence induction in a − 15° head-down tilt with full cervical spine extension reduced the amount of liquid aspirated but increased the difficulty in visualising the vocal cords and prolonged the time taken to intubate. As we cannot confirm the initial results with a patient-based study for ethical reasons, assessing the airway management in Trendelenburg position with full cervical spine extension in healthy patients without risk of aspiration might be a promising next step to take.

## Abbreviations

IQR: Interquartile range
RSI: Rapid sequence induction
